# Towards a better understanding of clinical disease activity scores in dogs with chronic enteropathies

**DOI:** 10.1080/01652176.2025.2573447

**Published:** 2025-11-03

**Authors:** Thomas Maufras, Tristan Méric, Elodie Darnis, Olivier Toulza, Chloé Arnould, Odile Sénécat, Cyril Duperrier-Simond, Loïc Desquilbet, Amandine Drut, Moez Rhimi, Juan Hernandez

**Affiliations:** a Centre Hospitalier Vétérinaire AniCura Aquivet, Internal Medicine Service, Mérignac, France; b Internal Medicine Service, Department of Clinical Sciences, ONIRIS VetAgroBio Nantes, Nantes, France; c Microbiota Interaction with Human and Animal (MIHA) team, Micalis Institute, Microbiology and Food Chain Départment, Institut National de Recherche Pour l’Agriculture, l’Alimentation et l’Environnement (INRAE), AgroParisTech, Université Paris-Saclay, Jouy‑en‑Josas, France; d Internal Medicine Service, Centre Hospitalier Vétérinaire Fregis, Gentilly, France; e Ecole Nationale Vétérinaire d’Alfort, IMRB, Maisons-Alfort, France

**Keywords:** Canine chronic inflammatory enteropathy, clinical scores, intra-observer, inter-observer, repeatability, reproducibility

## Abstract

The Canine Inflammatory Bowel Disease Activity Index (CIBDAI) and Canine Chronic Enteropathy Clinical Activity Index (CCECAI) are key tools for monitoring chronic enteropathies (CE) in dogs. Despite their widespread use, concerns persist regarding their intra-observer repeatability and inter-observer reproducibility, which may impact clinical and research applications. This study evaluated the reliability of these indices through a two-phase approach using anonymized clinical records. In Phase 1, two observers independently scored 41 consultation forms twice, one month apart, to assess repeatability and reproducibility. Phase 2 involved four observers with varying expertise who scored 59 forms using a standardized guide addressing Phase 1 inconsistencies. Statistical methods included Lin’s concordance correlation coefficient and Bland-Altman plots. High intra-observer repeatability was observed for most variables, but inter-observer reproducibility was limited for CIBDAI, CCECAI, and fluctuating parameters like stool consistency and defecation frequency. The standardized guide marginally improved consistency but did not resolve discrepancies. Expert evaluators did not consistently outperform non-experts. Reproducibility declined in more clinically severe cases. These findings highlight the need for standardized training, dynamic scoring systems, and digital tools to enhance reliability. Addressing these limitations is critical to improve clinical decision-making and research outcomes in canine CE.

## 1. Introduction

Chronic inflammatory enteropathies (CIE) are a group of diseases with an unclear origin, thought to arise from a combination of aberrant immune responses and disruptions in microbiota-host interactions in genetically predisposed individuals. One classification differentiates CIE based on the therapeutic response observed, categorizing cases as food-responsive (FRE), microbiota-modulation responsive (MrMRE), immunosuppressant-responsive (IRE) or non-responsive enteropathy (NRE) (Dupouy-Manescau et al. 2024). When associated with hypoalbuminemia, CIE merges into protein-losing enteropathies (PLE). Based on the response to treatment, PLEs may be subcategorized as either food-responsive PLEs (FR-PLE), immunosuppressant-responsive PLEs (IR-PLE) or non-responsive PLEs (NR-PLE). There is no curative treatment for CIE, and therapeutic efforts focus on alleviating the clinical severity of the disease.

Clinical monitoring remains essential and cannot be entirely replaced by biological parameters. The Canine Inflammatory Bowel Disease Activity Index (CIBDAI) and the Canine Chronic Enteropathy Clinical Activity Index (CCECAI) were developed and validated in 2003 and 2007, respectively (Allenspach et al. 2007; Jergens et al. 2003). These scores were initially designed as clinical tools to provide accurate, objective, and reproducible assessments of disease activity in dogs. For the CIBDAI, six variables were selected based on their correlation with objective laboratory and histological scores: attitude/activity, appetite, vomiting, stool consistency, stool frequency, and weight loss (Jergens et al. 2003). The CCECAI expanded on this by including three additional variables associated with prognosis: serum albumin concentration, pruritus, peripheral edema or ascites or both (Allenspach et al. 2007).

Both scores have been widely used to establish correlations between clinical activity and imaging findings, biological parameters, histological scores and prognosis (Heilmann et al. 2014; McMahon et al. 2010; Gaschen et al. 2008; Allenspach et al. 2019). Disease severity is categorized as follows: mildly active IBD or CIE (CIBDAI/CCECAI 4–5), moderate disease (CIBDAI/CCECAI 6–8), severe disease (CIBDAI ≥ 9, CCECAI 9–11), and very severe disease (CCECAI ≥ 12). These scores are also extensively used to validate clinical responses to dietary or medical treatments for dogs with chronic gastrointestinal signs (Nagata et al. 2020). In clinical trials, response to treatment is traditionally defined as a more than 50% reduction in the initial score or a final CIBDAI/CCECAI score below or equal to 3 (Allenspach et al. 2007; Jergens et al. 2003).

The variability of clinical scoring can influence therapeutic decision-making, eligibility for clinical trials, or the assessment of treatment effects (Nagata et al. 2020). This issue becomes particularly significant when multiple clinicians are involved in patient follow-up. Despite acknowledged usefulness of CIBDAI and CCECAI in the veterinary community, the intra-observer repeatability and inter-observer reproducibility of these scores has not been assessed. In the authors’ experience, it is observed that a substantial variation in CIBDAI and CCECAI scores assigned to the same clinical case by students, interns, residents, and senior clinicians. The inter-observer reproducibility of these scores was also a concern raised by authors of previous studies (Gori et al. 2022; Im Hof et al. 2012; Hanifeh et al. 2018; Heilmann et al. 2018; Kathrani et al. 2019). The aim of this study consequently was to evaluate the intra-observer repeatability and inter-observer reproducibility of CIBDAI and CCECAI scores in dogs with CE.

Evaluating intra-observer repeatability and inter-observer reproducibility in clinical scores presents inherent challenges. To assess inter-observer reproducibility, multiple clinicians would need to evaluate the same patient simultaneously under identical conditions to ensure a consistent clinical presentation. Similarly, assessing intra-observer repeatability would require the same clinician to reassess the same patient after a delay, during which clinical signs might naturally fluctuate. These approaches are not feasible due to ethical, logistical, and clinical considerations.

In this study, we addressed these challenges by using a highly detailed consultation form aimed at standardizing the collection of clinical information, regardless of the clinician. The objective of this work is firstly assessing the (intra-observer) repeatability and (inter-observer) reproducibility, and identifying potential sources of discrepancy. Then, evaluate the ability of specific guidelines in clarifying the scoring criteria identified as sources of variation during the pilot phase and subsequently improving repeatability and reproducibility.

## 2. Materials and methods

### 2.1. Study design

**Figure 1.**
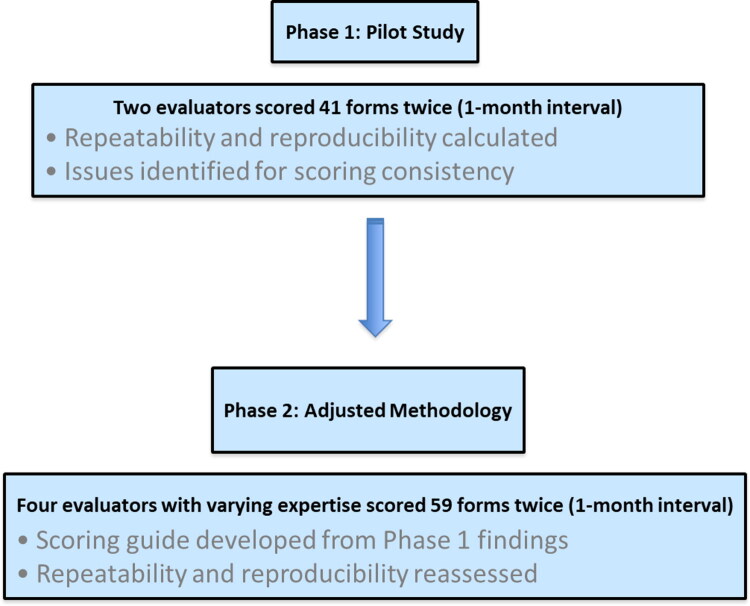
Visual representation of the study design.

The study was based on detailed and comprehensive clinical data collected during gastroenterology consultations at Oniris Veterinary Teaching Hospital (OVTH) using a consultation form specifically designed for this research (see Section 2.2). The work was divided into two successive phases (Figure 1):

- Phase 1 (Pilot Study): Anonymized consultation forms were reviewed by two evaluators, E1 and E2 (T. Méric and A. Drut), both affiliated with OVTH. The evaluators independently scored each variable required to calculate the CIBDAI and CCECAI scores. A second evaluation was conducted one month later, with the cases presented in a random order. Repeatability and reproducibility were then calculated. This phase will allow the identification variables influencing repeatability and reproducibility allowing discussions with the evaluators to analyze differences in interpretation.
- Phase 2: This phase utilized the same anonymized consultation forms as Phase 1 to evaluate repeatability and reproducibility among evaluators E3, E4, E5, and E6 (E. Darnis, O. Toulza, C. Boudenne, and A. Dauphin), who had varying levels of expertise and were not affiliated with OVTH. The assessment was guided by a scoring guide developed to standardize interpretation (see Section 2.5.1).

### 2.2. Consultation form

The consultation form was specifically designed for the purposes of this study by two of the authors (T. Méric and J. Hernandez) and was systematically used for all gastroenterology cases presented at OVTH (Appendix 1). The document provided detailed documentation of the variables required to calculate the CIBDAI and CCECAI scores, among other clinical data.

A significant section of the form was dedicated to describing the progression of clinical signs when they were inconsistent. This allowed for the detailed recording of variations occurring from day to day, or even within shorter intervals, such as during a single walk or defecation. For example, cases where stools were firm at the beginning of defecation but turned to diarrhea by the end could be thoroughly described and recorded. This level of detail ensured that the evaluators had the most comprehensive clinical information available.

To ensure impartiality and prevent any distinguishing features from helping evaluators recognize cases between the two readings, the content of the forms was transcribed into a spreadsheet for standardized presentation. Evaluators not working at authors’ institution were also recruited in Phase 2 of the study to further prevent recognition of cases based on reported clinical signs.

Multiple forms could pertain to the same animal, provided they were completed during different consultations. Under these conditions, we considered the two forms completed for the same animal as independent, as they were filled out by different team members (student, intern, resident) based on information collected during each visit, without access to the previous form. Therefore, the influence of one form on another was deemed negligible.

### 2.3. Clinical case selection

Dogs with CIE were recruited between May 1^st^ 2020 and May 1^st^ 2024 at the OVTH. In brief, the ­diagnosis of CIE was based on gastrointestinal clinical signs persisting for at least three weeks, after excluding extraintestinal diseases such as hepatic, pancreatic, or renal disorders, as well as infectious diseases and focal gastrointestinal wall diseases. Included cases were categorized based on their therapeutic response as food-responsive (FRE), microbiota modulation-responsive (MrMRE), immunosuppressant-responsive (IRE), any combination of these classifications, or nonresponsive enteropathy (NRE). MrMRE cases were defined as dogs showing marked clinical improvement following microbiota-targeted interventions, which included either fecal microbiota transplantation or administration of a probiotic containing at least five distinct bacterial strains at high viable counts (≥10^9^ CFU per strain per day), given for a minimum of two weeks. Both protein-losing and non-protein-losing cases were eligible for inclusion. Cases in which diagnostic evaluation revealed a neoplastic process were excluded. Cases for which the consultation form was not exhaustively filled also were not selected.

### 2.4. Phase 1: pilot study

The pilot phase involved presenting consultation forms of dogs diagnosed with CE to two evaluators, E1 and E2. Each evaluator was instructed to review the records and assign a score between 0 and 3 to each item, based on the criteria outlined in Table 1, extracted from the original publication.

**Table 1.** Scoring criteria for CIBDAI and CCECAI based on the original publications (2, 3).

Criterion	CIBDAI	CCECAI
Attitude/Activity	0 normal 1 slightly decreased 2 moderately decreased 3 severely decreased	0 normal 1 slightly decreased 2 moderately decreased 3 severely decreased
Appetite	0 normal 1 slightly decreased 2 moderately decreased 3 severely decreased	0 normal 1 slightly decreased 2 moderately decreased 3 severely decreased
Vomiting	0 normal 1 mild (1×/week) 2 moderate (2–3×/weed) 3 severe (>3/week)	0 normal 1 mild (1×/week) 2 moderate (2–3×/week) 3 severe (>3/week)
Stool consistency	0 normal 1 slightly soft feces 2 very soft feces 3 watery diarrhea	0 normal 1 slightly soft feces 2 very soft feces 3 watery diarrhea
Stool frequency	0 normal 1 slightly increased (2–3×/day) or fecal blood, mucus or both 2 moderately increased (4–5×/day) 3 severely increased (>5×/day)	0 normal 1 slightly increased (2–3×/day) or fecal blood, mucus or both 2 moderately increased (4–5×/day) 3 severely increased (>5×/day)
Weight Loss	0 none 1 mild (<5%) 2 moderate (5–10%) 3 severe (>10%)	0 none 1 mild (<5%) 2 moderate (5–10%) 3 severe (>10%)
Albumin levels		0 albumin >20 g/L* 1 albumin 15–19.9 g/L* 2 albumin 12–14.9 g/L* 3 albumin <12 g/L*
Ascites and peripheral edema		0 none 1 mild ascites or peripheral edema 2 moderate amount of ascites/peripheral edema 3 severe ascites/pleural effusion and peripheral edema
Pruritus		0 no pruritus 1 occasional episodes of itching 2 regular episodes if itching but stops when the dog is asleep 3 dog regularly wakes up because of itching

*Albumin value is indicated for a lower reference limit of 24 g/L.

To minimize potential bias due to fatigue or the influence of earlier cases on subsequent evaluations, evaluators were instructed to limit their reading sessions to a maximum of 1 h per day and to complete their assessments within 5 days. One month later, the same set of cases was presented to the evaluators in a different order, with identical instructions.

The CIBDAI and CCECAI scores were then compared between the two evaluators and between the two reading sessions. An analysis meeting involving the authors was subsequently conducted to review items that showed poor repeatability or reproducibility. The goal was to identify hypothetical underlying causes and develop a recommendation guide to standardize scoring practices.

### 2.5. Phase 2: adjusted methodology following the pilot study

#### 2.5.1. Guide to improve intra-observer repeatability and inter-observer reproducibility

To optimize repeatability and reproducibility, a scoring guide was developed to address the low repeatability or reproducibility for variables identified during the pilot phase (Table 2). In general, the assigned score should consider the evolution of the parameter since the previous consultation. Thus, changes in clinical signs were a key factor in building scores. For animals with relatively consistent signs during the evaluation period, each variable had to be scored according to the original scale, effectively averaging the observations over the specified time frame. The length of the evaluation period was left to the clinician’s discretion and depended on the interval between visits and the trend in clinical signs (e.g. for closely spaced visits, the interval between the previous and current visits might be appropriate). For animals with fluctuating signs, assigning a score at a single time point (a “snapshot”) was considered unreliable and unrepresentative of the true clinical situation. The recommendation was to assign an “averaged” score that accounted for the intensity, frequency, and progression of the signs over the evaluation period.

**Table 2.** Scoring guide developed to clarify points of disagreement identified during the pilot study.

Criterion	CIBDAI/CCECAI	Indications
Attitude/Activity	0 normal 1 slightly decreased 2 moderately decreased 3 severely decreased	Whenever possible, the score should reflect a deviation from the normal condition. The alteration must be attributable to the digestive disease and not to a concurrent condition
Appetite	0 normal 1 slightly decreased 2 moderately decreased 3 severely decreased	This encompasses the quantity of food ingested and any changes in eating patterns, such as increased meal duration or frequency, or selectivity for specific ingredients or types of food
Vomiting	0 normal 1 mild (1×/week) 2 moderate (2–3×/week) 3 severe (>3/week)	Rely solely on the provided numbers. For example, dogs vomiting less than once per week should be scored as 0, unless vomiting is the primary concern
Stool consistency	0 normal 1 slightly soft feces 2 very soft feces 3 watery diarrhea	Use the Purina Scale Fecal Score (**PSFS**) and assign scores based on the mean fecal score. A score of 0 corresponds to a **PSFS** of 1–2, 1 corresponds to a **PSFS** of 3–4, 2 corresponds to a **PSFS** of 5–6, and 3 corresponds to a **PSFS** of 7
Stool frequency	0 normal 1 slightly increased (2–3×/day) or fecal blood, mucus or both 2 moderately increased (4–5×/day) 3 severely increased (>5×/day)	Base the evaluation solely on the provided numerical data
Weight Loss	0 none 1 mild (<5%) 2 moderate (5–10%) 3 severe (>10%)	Estimate the percentage of weight loss attributable to the disease by considering the difference between the current body weight and the weight prior to the onset of gastrointestinal clinical signs. Take into account the current body condition score, the degree of muscle atrophy, the presence of ascites or peripheral edema (which may falsely elevate the current body weight), and any potential caloric intake restriction implemented by the owner (which may falsely lower the body weight)

For any clinical sign that was considered a problem for the animal, the minimum score was 1. Only integer numbers (0, 1, 2, or 3) could be used.

#### 2.5.2. Evaluators

The evaluators E3, E4, E5, and E6 were selected based on two levels of expertise: two internal medicine board-certified specialists (E3 and E4) formed the “expert” group, while two general practitioners (E5 and E6) formed the “non-expert” group.

At T1, all these four evaluators received the following materials: a spreadsheet containing the clinical information, an interpretation guide for each item, a blank spreadsheet to record the assigned scores, and detailed reading instructions (sessions limited to 1 h maximum, with a total completion time of 7 days).

At T2 (one month later), the same materials were provided, except that the spreadsheet of clinical cases was reorganized into a random order.

### 2.6. Repeatability and reproducibility analyses

The repeatability of the CIBDAI and CCECAI scores was evaluated for each observer by comparing their results from the first measurements (at T1) and the second measurements (at T2) using the same methods. The reproducibility of the CIBDAI and CCECAI scores between evaluators was assessed by comparing the sets of results from the initial measurements (at T1). The Lin’s concordance correlation coefficient (CCC) (Lin et al. 2007) and the Bland and Altman (B&A) graphic method (Bland and Altman 1999) were used to quantify repeatability and reproducibility. Each CCC was provided with its 95% confidence interval (CI). The mean difference between two sets of measurements in the B&A method (named “bias”) was provided with its 95%CI. Limits of agreement (LoA) provided by the B&A method were 95% LoA, and they were provided with their 95%CI.

Two sets of measurements were deemed concordant if CCC was greater than 0.81 (interpreted as “very good” by the Landis and Koch’s classification, and as “rather good” by the Partik’s classification) and both following B&A criteria were met (Mantha et al. 2000; Desquilbet 2023):

1. The “bias” between the two sets of measurements should not exceed ±1 unit.
2. The LoA fall within ±1 unit for isolated variables and ±2 units for CIBDAI and CCECAI, defined as the “limits of agreement.”

To have 80% chances to demonstrate a minimum CCC of 0.81, estimating that the expected CCC would be at least equal to 0.91, the number of consultation forms to review per observer was 40 (Excel^®^ file for calculating the required number of dogs provided by Loic Desquilbet) (Desquilbet 2023; Walter et al. 1998). At least 40 consultation forms were planned to be collected during the pilot study, and additional forms were included in the second phase of the study to increase statistical power.

To explore whether disease severity influence scoring consistency, we performed a subgroup analysis of intra-observer repeatability and inter-observer reproducibility stratified by clinical score severity. For each score (CIBDAI and CCECAI), cases were categorized into two groups based on the mean score obtained across all evaluators and timepoints. For CIBDAI: low-severity group with mean CIBDAI < 5.5 and high-severity group with mean CIBDAI ≥ 5. For CCECAI: low-severity group with mean CCECAI < 7 and high-severity group with mean CCECAI ≥ 7. These thresholds were selected based on published disease severity categories described in the original validation studies for these indices (Allenspach et al. 2007; Jergens et al. 2003). Specifically, a CIBDAI score ≤5 corresponds to clinically insignificant to mild disease, and ≥6 to moderate to severe disease; CCECAI score ≤5 indicates clinically insignificant to mild disease, between 6 and 8 moderate disease, and ≥7 severe to very severe disease. Within each severity subgroup, repeatability and reproducibility were reassessed using Lin’s CCC and B&A analyses.

## 3. Results

### 3.1. Pilot study results

#### 3.1.1. Study sample

A total of 31 dogs provided 41 consultation forms, with four dogs contributing two forms each, one dog contributing three forms, and another contributing five forms. The median age of the dogs was 6 years (interquartile range: 2.5–9.5 years).

The cohort included 11 intact males (36%), 10 neutered males (32%), 9 spayed females (29%), and 1 intact female (3%). The most commonly represented breeds were Yorkshire Terriers (4/31, 13%), Golden Retrievers (4/31, 13%), Shih Tzus (2/31), Bernese Mountain Dogs (2/31), and Australian Shepherds (2/31), with various other breeds represented by a single individual each.

Among the recruited cases, 7 dogs were classified as FRE, 11 as Mr-MRE, 12 as IRE, and 1 as NRE. CIBDAI and CCECAI scores ranged from 0 to 11 (mean: 5, interquartile range: 4–7) and 0 to 16 (mean: 6, interquartile range: 4–8), respectively.

#### 3.1.2. Repeatability

A total of 41 consultation forms were completed. The results of the statistical analysis evaluating repeatability are summarized in Supplementary File 1. Both observers demonstrated a satisfactory repeatability in assessing CIBDAI, CCECAI, activity, appetite, vomiting, frequency of defecation, weight loss, abdominal fluid, edema, and pruritus. E1 was also repeatable in assessing fecal consistency, whereas E2 was not (CCC: 0.87 [0.76; 0.93]; bias: 0.05 [−0.12; 0.22]; lower LOA: −1.02 [−1.32; −0.72]; upper LOA: 1.12 [0.82; 1.42]).

#### 3.1.3. Reproducibility

Results of the statistical analysis performed to assess the reproducibility are summarized in Supplementary File 2. E1 and E2 demonstrated a satisfactory reproducibility in assessing activity, appetite, vomiting, abdominal fluid and edema, and pruritus. However, they were not reproducible in assessing CIBDAI, CCECAI, fecal consistency, frequency of defecation, and weight loss (Table 3).

**Table 3.** Selected parameters that did not meet the reproducibility criteria in the pilot study. Full analyses are presented in Supplementary File 2.

Score	Lin’s concordance coefficient	Bias	Lower 95% LoA	Upper 95% LoA	Agreement
CIBDAI	0.88 [0.78; 0.93]	0.17 [−0.26; 0.60]	−2.49 [−3.25; −1.74]	2.83 [2.08; 3.59]	No
CCECAI	0.91 [0.83; 0.95]	0.049 [−0.386; 0.484]	−2.651 [−3.416; −1.886]	2.749 [1.984; 3.513]	No
Fecal consistency	0.85 [0.73; 0.92]	0.15 [−0.03; 0.33]	−0.98 [−1.29; −0.66]	1.27 [0.95; 1.59]	No
Frequency of defecation	0.86 [0.75; 0.92]	−0.20 [−0.36; −0.03]	−1.20 [−1.48; −0.91]	0.81 [0.52; 1.09]	No
Weight loss	0.80 [0.66; 0.89]	0.07 [−0.15; 0.3]	−1.34 [−1.74; −0.94]	1.49 [1.09; 1.89]	No

### 3.2. Reviewing of worksheets items that were source of disagreement

E1 and E2 reviewed 30 out of 41 consultation forms (73%) that were identified as sources of disagreement.

#### 3.2.1. Fecal consistency

In consultation forms indicating cyclic clinical signs, one observer scored the item based on the highest reported fecal score, while the other estimated an average fecal score over the preceding weeks. To address this lack of reproducibility, evaluators in the second phase of the study were instructed to use the mean fecal score based on the Purina Scale (PSFS), as described in the consultation forms, and translate it into the corresponding CIBDAI item scores: score 0 for a PSFS of 1 or 2, score 1 for a PSFS of 3 or 4, score 2 for a PSFS of 5 or 6, score 3 for a PSFS of 7.

#### 3.2.2. Frequency of defecation

For cases with cyclic clinical signs, one observer scored the item based on the highest frequency of defecation reported, while the other estimated an average frequency over the past few weeks. Additionally, one observer considered twice-daily defecation to be normal and assigned a score of 0, whereas the other adhered to the instructions from the original CIBDAI study, which treated this as abnormal and also assigned a score of 1. To address these discrepancies, the instructions for the second phase of the study guided evaluators to calculate an average defecation frequency and to rely strictly on the numerical data provided in the original publications (Table 1).

#### 3.2.3. Weight loss

One observer scored weight loss solely based on the difference between bodyweight recorded before the onset of clinical signs and at the time of consultation. In contrast, the other observer incorporated additional factors, including body condition score, muscle atrophy, presence of abdominal fluid or peripheral edema, and potential caloric intake restrictions imposed by the owner, alongside the weight measurements.

To reduce this source of discrepancy, instructions in the second phase of the study guided evaluators to estimate the percentage of weight loss attributable to the disease. This required consideration of several factors, including the difference between the current bodyweight and the preclinical weight, the extent of current muscle atrophy, the presence of ascites or peripheral edema (which could falsely increase bodyweight), and any caloric intake restriction voluntarily applied by the owner (which could falsely decrease live weight).

### 3.3. Phase 2 results

#### 3.3.1. Study sample

**Figure 2.**
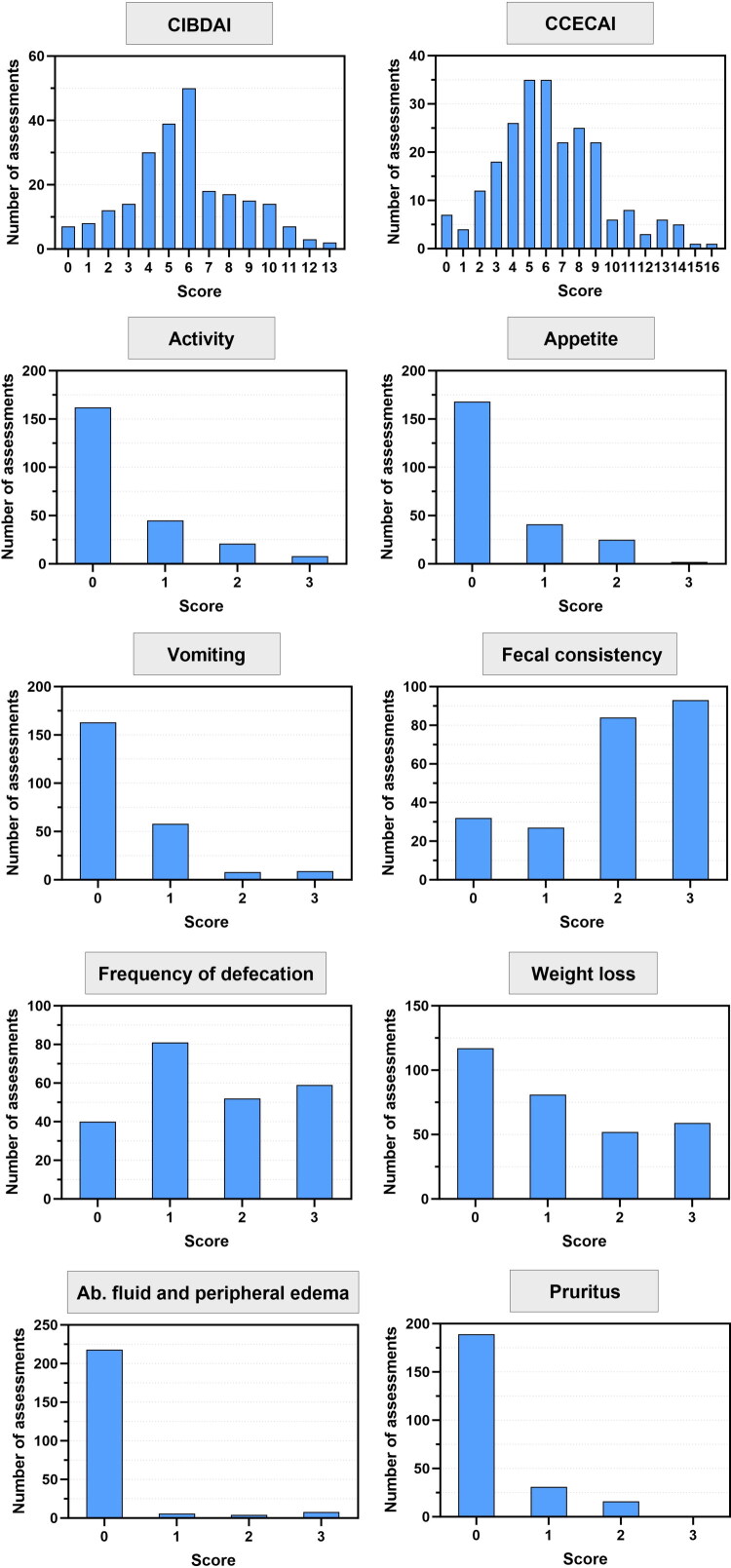
Distribution of CIBDAI scores and individual variables (sum of every assessment of each observer at T1).

A total of 49 dogs provided 59 consultation forms, with four dogs contributing two forms each, one dog contributing three forms, and another contributing five forms. The median age of the dogs was 4 years (interquartile range: 1–8.5 years). The cohort included 18 intact males (37%), 14 neutered males (29%), 13 spayed females (27%), and 4 intact females (8%). The most commonly represented breeds were Yorkshire Terriers (7/49, 14%), Golden Retrievers (7/49, 14%), Bernese Mountain Dogs (3/49), Shih Tzus (2/49), Australian Shepherds (2/49) and Labrador Retrievers (2/49), with various other breeds represented by a single individual each. Among the recruited cases, 14 dogs were classified as FRE, 14 as Mr-MRE, 19 as IRE, and 2 as NRE. Distribution of CCECAI, CIBDAI and each item score is depicted in Figure 2. CIBDAI and CCECAI scores ranged from 0 to 13 (mean: 6, interquartile range: 4–7) and 0 to 16 (mean: 6, interquartile range: 4–8), respectively.

#### 3.3.2. Repeatability

**Figure 3.**
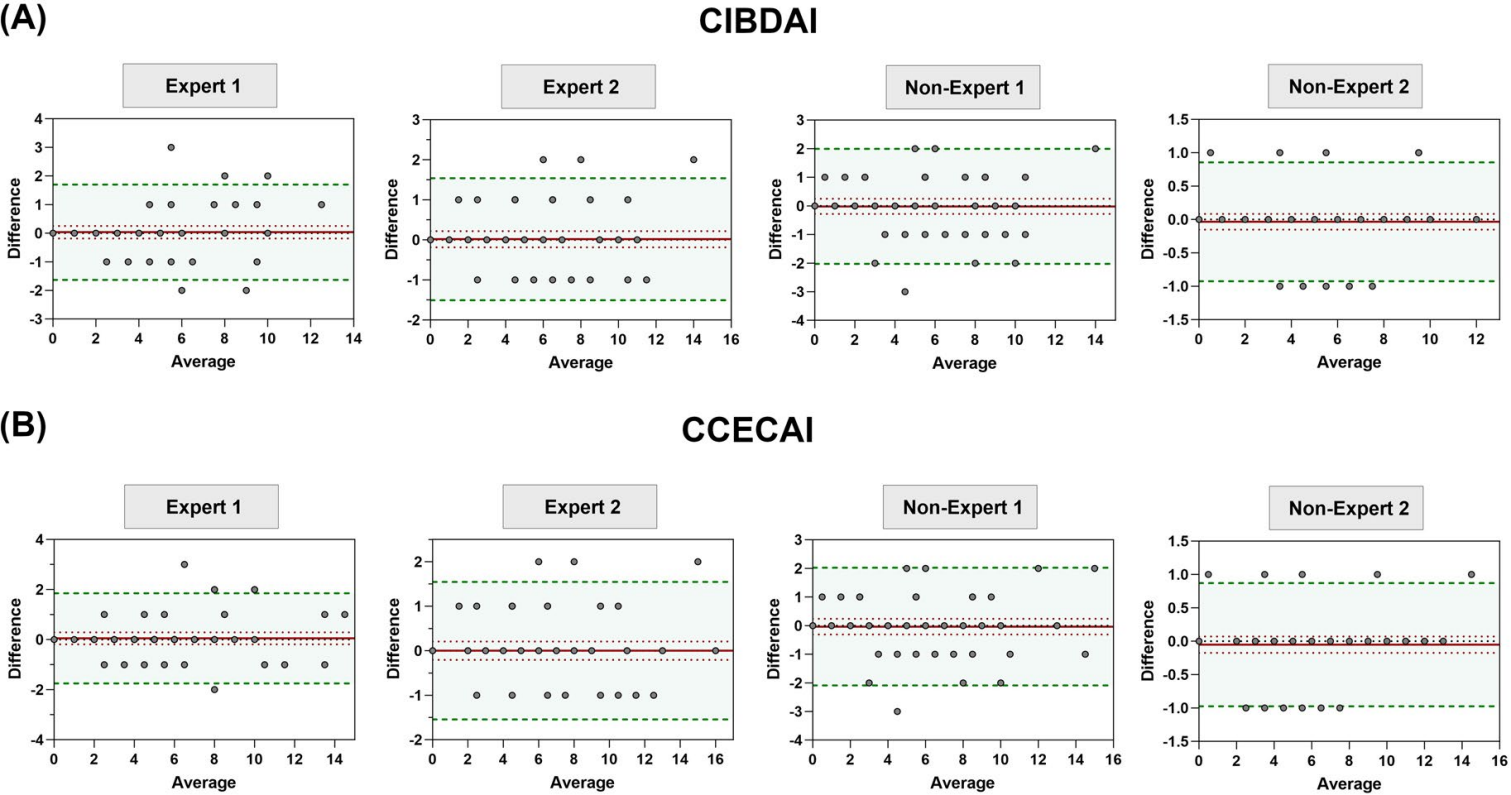
Bland-Altman Plots illustrating the repeatability of CIBDAI (A) and CCECAI (B) scores for each observer. The red line represents the bias, which should not exceed ±1 unit. The green lines indicate the lower and upper limits of agreement, which should fall within ±2 units for CIBDAI and CCECAI scores. Each plot demonstrates the level of agreement for the respective scores, highlighting areas where measurements deviate from the bias and agreement thresholds. Non-Expert 1 did not achieve repeatability criteria for CIBDAI and CCECAI scores.

A total of 59 consultation forms were completed. The results of the statistical analysis evaluating repeatability are summarized in Supplementary File 3. The Bland-Altman graphical representation for repeatability of the CIBDAI and CCECAI scores is presented in Figure 3. Three observers (Expert 1 and 2, and Non-expert 2) demonstrated a satisfactory repeatability in assessing the CIBDAI, CCECAI, and most individual variable. However, Non-expert 1 did not achieve repeatability criteria for the vomiting and frequency of defecation parameters, as well as for the CIBDAI and CCECAI scores (Table 4).

**Table 4.** Selected parameters that did not meet the repeatability criteria for non-expert 1 and expert 1. Full analyses are presented in Supplementary File 3.

Score	Observer	Lin’s concordance coefficient	Bias	Lower 95% LoA	Upper 95% LoA	Agreement
CIBDAI	Non-expert 1	0.93 [0.89; 0.96]	−0.017 [−0.284; 0.25]	−2.027 [−2.492; −1.561]	1.993 [1.527; 2.459]	No
CCECAI	Non-expert 1	0.95 [0.91; 0.97]	−0.034 [−0.308; 0.24]	−2.092 [−2.569; −1.615]	2.024 [1.547; 2.501]	No
Vomiting	Non-expert 1	0.75 [0.61; 0.84]	−0.119 [−0.265; 0.027]	−1.216 [−1.47; −0.961]	0.978 [0.724; 1.233]	No
Frequency of defecation	Non-expert 1	0.85 [0.76; 0.91]	0.224 [0.076; 0.372]	−0.88 [−1.138; −0.622]	1.328 [1.07; 1.586]	No
Weight loss	Non-expert 1	0.85 [0.76; 0.91]	0.224 [0.076; 0.372]	−0.88 [−1.138; −0.622]	1.328 [1.07; 1.586]	No
Pruritus	Expert 1	0.8 [0.69; 0.88]	0 [−0.097; 0.097]	−0.728 [−0.897; −0.559]	0.728 [0.559; 0.897]	No

#### 3.3.3. Reproducibility

**Figure 4.**
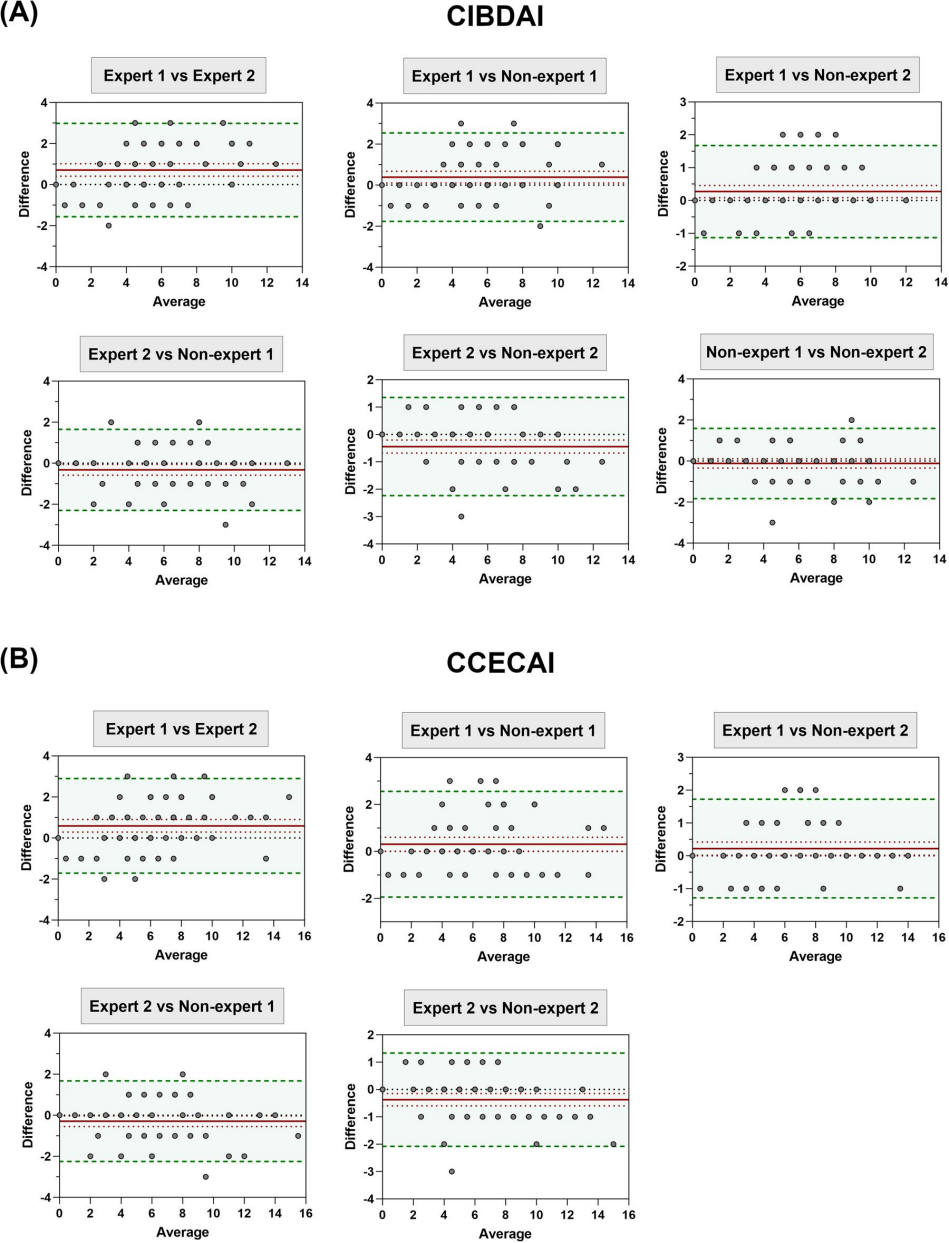
Bland-Altman Plots illustrating the reproducibility of CIBDAI (A) and CCECAI (B) scores for various pairwise comparisons of observers. The red line represents the bias, which should not exceed ±1 unit. The green solid lines indicate the lower and upper limits of agreement, which should fall within ±2 units for CIBDAI and CCECAI scores. The plots highlight satisfactory reproducibility between certain observer pairs, such as Expert 1 and Non-expert 2, and Non-Expert 1 and Non-Expert 2, for both CIBDAI and CCECAI scores. In contrast, other comparisons, such as Expert 1 *versus* Expert 2, demonstrate non-satisfactory reproducibility.

Results of the statistical analysis performed to assess the reproducibility are summarized in Supplementary File 4. The Bland-Altman graphical representation for reproducibility of the CIBDAI and CCECAI scores is presented in Figure 4. For CIBDAI, a satisfactory reproducibility was achieved between Expert 1 and Non-Expert 2 (CCC: 0.96 [0.93; 0.97]; bias: 0.271 [0.085; 0.458]; lower LoA: −1.130 [−1.455; −0.806]; upper LoA: 1.673 [1.348; 1.998]) and between Non-Expert 1 and Non-Expert 2 (CCC: 0.95 [0.91; 0.97]; bias: −0.119 [−0.346; 0.109]; lower LoA: −1.829 [−2.225; −1.433]; upper LOA: 1.592 [1.195; 1.988]). However, reproducibility for other pairwise comparisons, such as Expert 2 versus Non-Expert 1, was not satisfactory (CCC: 0.93 [0.89; 0.96]; bias: −0.322 [−0.585; −0.059]; lower LoA: −2.297 [−2.754; −1.839]; upper LoA: 1.653 [1.11; 1.95]).

For CCECAI, a satisfactory reproducibility was achieved between Expert 1 and Non-Expert 2 (CCC: 0.97 [0.94; 0.98]; bias: 0.220 [0.02; 0.42]; lower LoA: −1.283 [−1.632; −0.935]; upper LoA: 1.724 [1.375; 2.072]) and between Non-Expert 1 and Non-Expert 2 (CCC: 0.96 [0.94; 0.98]; bias: −0.085 [−0.313; 0.144]; lower LoA: −1.803 [−2.201; −1.405]; upper LoA: 1.634 [1.235; 2.032]). Other comparisons showed non-satisfactory reproducibility, including Expert 1 versus Expert 2 (CCC: 0.92 [0.86; 0.95]; bias: 0.5932 [0.287; 09]; lower LoA: −1.712 [−2.246; −1.178]; upper LoA: 2.899 [2.364; 3.433]).

For individual variables, high reproducibility was consistently observed across all comparisons for activity, appetite and abdominal fluid, with CCC ranging from 0.84 to 0.99. In contrast, reproducibility criteria were only partially achieved for vomiting, fecal consistency, frequency of defecation, weight loss and pruritus. Comparisons involving experts did not demonstrate better reproducibility compared to those involving non-experts.

#### 3.3.4. Association between disease severity and scoring agreement

Across both low and high severity subgroups, intra-observer repeatability was generally good, though with some variability among observers and scores (Supplementary File 5). Lin’s CCC values were consistently above 0.83, and most exceeded 0.90. For CIBDAI, agreement was achieved in 3 out of 4 observer combinations in the low activity group, and in 2 out of 4 in the high activity group. For example, Non-expert 1 showed excellent repeatability (CCC: 0.95 [0.88; 0.98]; bias: −0.115 [−0.29; 0.059]; lower LoA: −0.961 [−1.273; −0.649]; upper LoA: 0.730 [0.418; 1.042]), whereas Expert 2 did not meet the predefined agreement thresholds despite a relatively high CCC (CCC: 0.83 [0.66; 0.92]; bias: −0.077 [−0.488; 0.334]; lower LoA: −2.070 [–2.805; −1.334]; upper LoA: 1.916 [1.181; 2.651]). For CCECAI, 3 out of 4 observer combinations met the agreement criteria in the low activity group, and 2 out of 4 in the high activity group. In the high CCECAI group, for instance, Expert 1 displayed strong repeatability (CCC: 0.93 [0.84; 0.97]; bias: −0.130 [−0.528; 0.267]; lower LoA: −1.933 [−2.650; −1.216]; upper LoA: 1.672 [0.955; 2.389]), while Expert 2 failed to meet the agreement criteria (CCC: 0.89 [0.77; 0.95]; bias: −0.130 [−0.588; 0.327]; lower LoA: −2.203 [−3.028; −1.379]; upper LoA: 1.942 [1.118; 2.767]).

In contrast to intra-observer repeatability, inter-observer reproducibility was more variable and appeared to be more affected by disease severity (Supplementary File 6). In the low-CIBDAI subgroup, agreement was observed in 3 out of 6 pairs of evaluators (50%). In contrast, only 2 pairs (33%) met agreement criteria in the high*-*CIBDAI subgroup. For the CCECAI, 4 out of 6 pairs (67%) showed agreement in the low-activity subgroup, whereas no pair fulfilled agreement criteria in the high-CCECAI subgroup.

## 4. Discussion

Repeatability and reproducibility in clinical scoring can significantly impact the reliability of clinical trials and decision-making in veterinary practice. For instance, a cutoff score of 8 for the CCECAI has demonstrated good sensitivity (83%) and specificity (89%) in distinguishing protein-losing enteropathies (PLE) that are likely to respond to dietary changes from those that are not (Nagata et al. 2020). However, reproducibility in the CCECAI score can shift an animal from one category to another, directly influencing therapeutic decisions, especially in borderline cases. Reproducibility also raises concerns in clinical research protocols involving multiple clinicians, as the absence of scientifically validated reproducibility data introduces uncertainty (Gori et al. 2022).

Our results provide some evidence that CCECAI and CIBDAI scores were generally repeatable (among three out of four observers) but lacked reproducibility between observers when assessed using a consultation form containing extensive information about gastrointestinal clinical signs.

This finding underscores the importance of involving a single clinician throughout an animal’s follow-up to ensure consistent assessments. The lack of reproducibility highlights differences in the interpretation of clinical signs among clinicians. In fact, during the pilot study, we identified that items reflecting variables prone to fluctuations due to disease progression such as stool consistency, frequency of defecation, and weight changes were the most affected by poor reproducibility. To address this, we proposed a scoring guide to help observers account for these fluctuations. These guidelines emphasized that scores should reflect the clinical evolution reported by the owner between evaluations. However, despite these measures, the inclusion of these instructions did not improve reproducibility for CIBDAI, CCECAI, stool consistency, frequency of defecation, or weight loss. Providing precise recommendations for the evaluation period remains challenging, particularly for animals with highly variable signs, which can fluctuate even within the same day or walk.

Similarly, in humans, the Crohn’s Disease Activity Index (CDAI) widely used to monitor patients with Crohn’s disease addresses daily fluctuations by collecting data over the preceding seven days. The CDAI integrates eight weighted variables, including the number of liquid or soft stools per day, abdominal pain, general well-being, complications, medication use, palpable abdominal masses, hematocrit levels, and weight changes (Best et al. 1976). Although effective, the CDAI has been criticized for subjectivity in some variables. For instance, one study found that, in patients with a CDAI above 150, 72% of the total score was derived from subjective components such as self-reported symptoms and general well-being. The authors suggested that distinguishing between subjective variables (e.g. symptoms and well-being) and objective measures (e.g. endoscopy, inflammatory markers) could improve the assessment of disease activity in Crohn’s disease clinical trials (Freeman 2008). Despite these limitations, its ability to account for clinical fluctuations provides a more comprehensive assessment compared to scores like the Harvey-Bradshaw Index, which relies solely on variables collected during a single consultation (Harvey and Bradshaw 1980).

Our study also highlights the potential influence of disease severity on scoring consistency. While intra-observer repeatability remained generally good across both low and high activity groups, inter-observer reproducibility was more affected by clinical severity. Good agreement between observers was more frequently achieved in animals with mild clinical signs than in those with more severe presentations. This may reflect the nature of low CIBDAI and CCECAI scores, which likely include a higher proportion of zero ratings, indicating the absence of abnormalities for several criteria. These “default” scores are easier to assign and inherently more reproducible, thereby facilitating agreement between observers. These findings suggest that reproducibility is not only observer-dependent, but also context-dependent, with scoring discrepancies becoming more likely in clinically complex cases. Such variability reinforces the need for refined scoring systems that integrate longitudinal data, better capture fluctuating signs, and offer clearer guidelines for evaluating severe presentations. Additionally, our results emphasize the value of assigning a single clinician to follow individual patients in clinical trials, especially those with moderate-to-severe disease. Likely, the “snapshot” nature of CIBDAI and CCECAI makes them less reliable for evaluating animals with fluctuating clinical signs. Developing a scoring system that incorporates daily ratings over a defined period, similar to the CDAI, could improve the accuracy of clinical assessments and case monitoring. The complexity of such an index could be mitigated using smartphone applications for daily data collection. Furthermore, subjective evaluations, such as stool consistency, could benefit from artificial intelligence (AI) and image recognition technologies. This technology has already proven effective in various fields of human medicine, such as medical imaging for detecting specific lesions (Khan et al. 2023), cytogenetics for automated karyotype classification (Khazaei et al. 2022), histopathology for identifying cancerous lesions (Peyret et al. 2023), ophthalmology for screening diabetic retinopathies (Quellec et al. 2017) and gastroenterology. For example, a convolutional neural network (CNN) was able to grade the endoscopic severity of ulcerative colitis in humans with accuracy comparable to that of experienced reviewers (Stidham et al. 2019). The integration of machine-readable data, input daily by pet owners through specially designed digital applications, could improve intra-observer repeatability and inter-observer reproducibility and provide a more dynamic view of clinical sign evolution compared to the retrospective collection of anamnestic data during consultations. Such tools could also involve pet owners more actively in their animal’s monitoring, improving data consistency and overall engagement.

Our study also explored the differences in scoring agreement between experts and non-experts. Expertise did not guarantee higher reproducibility, indicating that experience may not significantly influence scoring reliability. This finding suggests that standardized training for all clinicians, regardless of expertise level, could enhance scoring consistency and reduce reproducibility.

In the second phase of the study, 21 out of 28 measurement sets were categorized as non-concordant solely because the limits of agreement defined by the B&A method exceeded the pre-established acceptable thresholds. To ensure rigorous assessment, we opted for narrower limits of agreement (±1 for isolated variables and ±2 for CIBDAI and CCECAI), prioritizing the stringent expectations of most readers. However, broader limits of agreement might still be clinically acceptable to some practitioners. If a threshold of ±1.5 for isolated variables and ±2.5 for CIBDAI and CCECAI were deemed sufficient, only 11 measurement sets would remain non-concordant. Extending the limits further, to ±2 for isolated variables and ±3 for CIBDAI and CCECAI, would reduce the number of non-concordant sets to 6. Under this latter configuration, CIBDAI and CCECAI would be considered repeatable and reproducible.

It’s worth noting that the primary limitation of our study is that observer evaluations were based on forms transcribing consultation data rather than direct clinical evaluations. This approach ensured consistent material for analysis but eliminated the cognitive bias associated with the clinician-owner relationship. It is possible that the initial transcription of clinical data was influenced by the clinician conducting the consultation, potentially affecting observer interpretations. An additional limitation is that the item “abdominal fluid and peripheral edema” was most of the time given a score equal 0, preventing assessment of its repeatability and reproducibility. In addition, our study design did not allow for within-group comparisons to evaluate the direct impact of the scoring guideline on inter-observer reproducibility. Because the observers involved in Phase 1 (E1 and E2) differed from those in Phase 2 (E3–E6), it was not possible to assess whether the guideline improved scoring consistency within the same group of evaluators. Future studies should address this point by using the same observer cohort before and after implementation of such guidelines to better quantify their effectiveness.

## 5. Conclusion

As a conclusion, intra-observer repeatability and inter-observer reproducibility in clinical scoring instruments like CIBDAI and CCECAI has significant implications for both clinical practice and research. Our findings highlight the need for more reliable and consistent scoring methods that account for fluctuating clinical signs in canine CIE. Future research should focus on dynamic scoring systems that integrate longitudinal data and digital tools for daily symptom tracking. Standardizing clinician training and incorporating technological advancements, such as AI, could further enhance scoring accuracy and reliability. Addressing these challenges would improve follow-up precision and optimize treatment strategies.

## Supplementary Material

Appendix 1.xlsx

Supplementary file 5.docx

Supplementary file 6.docx

Supplementary file 1.docx

Supplementary file 4_AJ.docx

Supplementary file 3_AJ.docx

Supplementary file 2.docx

## Compliance with regulations and data management

This study was conducted in strict compliance with the European Union’s General Data Protection Regulation (GDPR, EU 2016/679). All medical data used in this retrospective study were derived exclusively from records of owners who had explicitly provided written consent for the use of their animals’ medical data for research purposes.
