# Supplementary material for: Towards a better understanding of clinical disease activity scores in dogs with chronic enteropathies
Source: Vet Q. 2025 Nov 3;45(1):2573447. doi: 10.1080/01652176.2025.2573447 (PMC12587788; doi:10.1080/01652176.2025.2573447)
Supplement: Supplementary file 5.docx [file TVEQ_A_2573447_SM4360.docx]

**Supplementary file 5**. Study of the **intra-observer repeatability of CIBDAI and CCECAI scores in dogs with low or high disease activity.** Repeatability was assessed for each observer using Lin’s CCC, B&A bias, and 95% LoA (limit of agreement), stratified by score type (low *v*s high).

| **Score** | **Observer** | **Lin's concordance coefficient** | **Bias** | **Lower 95% LOA** | **Upper 95% LOA** | **Agreement** |
| --- | --- | --- | --- | --- | --- | --- |
| Low CIBDAI | Expert 1 | 0,94 [0,87 ; 0,98] | 0,115 [-0,122 ; 0,353] | -1,038 [-1,463 ; -0,612] | 1,269 [0,843 ; 1,694] | Yes |
|  | Expert 2 | 0,83 [0,66 ; 0,92] | -0,077 [-0,488 ; 0,334] | -2,07 [-2,805 ; -1,334] | 1,916 [1,181 ; 2,651] | No |
|  | Non-expert 1 | 0,95 [0,88 ; 0,98] | -0,115 [-0,29 ; 0,059] | -0,961 [-1,273 ; -0,649] | 0,73 [0,418 ; 1,042] | Yes |
|  | Non-expert 2 | 0,95 [0,88 ; 0,98] | -0,077 [-0,272 ; 0,118] | -1,025 [-1,374 ; -0,675] | 0,871 [0,521 ; 1,221] | Yes |
| High CIBDAI | Expert 1 | 0,92 [0,84 ; 0,96] | -0,061 [-0,379 ; 0,258] | -1,823 [-2,388 ; -1,258] | 1,702 [1,137 ; 2,267] | Yes |
|  | Expert 2 | 0,87 [0,75 ; 0,93] | 0,03 [-0,34 ; 0,401] | -2,019 [-2,675 ; -1,362] | 2,079 [1,422 ; 2,736] | No |
|  | Non-expert 1 | 0,85 [0,71 ; 0,92] | 0,152 [-0,226 ; 0,529] | -1,934 [-2,603 ; -1,266] | 2,237 [1,569 ; 2,906] | No |
|  | Non-expert 2 | 0,849 [0,577 ; 1,121] | 0,97 [0,94 ; 0,99] | 0 [-0,154 ; 0,154] | -0,849 [-1,121 ; -0,577] | Yes |
| Low CCECAI | Expert 1 | 0,93 [0,86 ; 0,96] | 0,083 [-0,151 ; 0,317] | -1,273 [-1,686 ; -0,859] | 1,439 [1,026 ; 1,853] | Yes |
|  | Expert 2 | 0,84 [0,71 ; 0,92] | 0,028 [-0,329 ; 0,385] | -2,04 [-2,671 ; -1,41] | 2,096 [1,465 ; 2,727] | No |
|  | Non-expert 1 | 0,9 [0,81 ; 0,95] | -0,056 [-0,311 ; 0,2] | -1,533 [-1,984 ; -1,083] | 1,422 [0,971 ; 1,872] | Yes |
|  | Non-expert 2 | 0,95 [0,91 ; 0,98] | -0,111 [-0,288 ; 0,066] | -1,135 [-1,447 ; -0,823] | 0,913 [0,601 ; 1,225] | Yes |
| High CCECAI | Expert 1 | 0,93 [0,84 ; 0,97] | -0,13 [-0,528 ; 0,267] | -1,933 [-2,65 ; -1,216] | 1,672 [0,955 ; 2,389] | Yes |
|  | Expert 2 | 0,89 [0,77 ; 0,95] | -0,13 [-0,588 ; 0,327] | -2,203 [-3,028 ; -1,379] | 1,942 [1,118 ; 2,767] | No |
|  | Non-expert 1 | 0,89 [0,76 ; 0,96] | 0,217 [-0,27 ; 0,704] | -1,99 [-2,868 ; -1,112] | 2,425 [1,547 ; 3,303] | No |
|  | Non-expert 2 | 0,98 [0,96 ; 0,99] | 0,043 [-0,115 ; 0,202] | -0,675 [-0,961 ; -0,389] | 0,762 [0,476 ; 1,048] | Yes |
