# Supplementary material for: Towards a better understanding of clinical disease activity scores in dogs with chronic enteropathies
Source: Vet Q. 2025 Nov 3;45(1):2573447. doi: 10.1080/01652176.2025.2573447 (PMC12587788; doi:10.1080/01652176.2025.2573447)
Supplement: Supplementary file 6.docx [file TVEQ_A_2573447_SM4359.docx]

**Supplementary file 6**. Study of inter-observer reproducibility of CIBDAI and CCECAI scores in subgroups of low and high clinical activity. Reproducibility was assessed for each observer using Lin’s CCC, B&A bias, and 95% LoA (limit of agreement), stratified by score type (low *v*s high).

| **Score** | **Observer** | **Lin's concordance coefficient** | **Bias** | **Lower 95% LoA** | **Upper 95% LoA** | **Agreement** |
| --- | --- | --- | --- | --- | --- | --- |
| Low CIBDAI | Expert 1 vs Expert 2 | 0,88 [0,74 ; 0,95] | -0,154 [-0,51 ; 0,202] | -1,88 [-2,517 ; -1,243] | 1,572 [0,935 ; 2,209] | Yes |
|  | Expert 1 vs Non-expert 1 | 0,74 [0,5 ; 0,87] | 0,385 [-0,059 ; 0,828] | -1,768 [-2,562 ; -0,974] | 2,537 [1,743 ; 3,331] | No |
|  | Expert 1 vs Non-expert 2 | 0,85 [0,68 ; 0,93] | -0,308 [-0,665 ; 0,049] | -2,04 [-2,68 ; -1,401] | 1,425 [0,786 ; 2,064] | No |
|  | Expert 2 vs Non-expert 1 | 0,79 [0,61 ; 0,89] | 0,231 [-0,186 ; 0,648] | -1,792 [-2,538 ; -1,045] | 2,253 [1,507 ; 2,999] | No |
|  | Expert 2 vs Non-expert 2 | 0,86 [0,72 ; 0,94] | -0,154 [-0,51 ; 0,202] | -1,88 [-2,517 ; -1,243] | 1,572 [0,935 ; 2,209] | Yes |
|  | Non-expert 1 vs Non-expert 2 | 0,92 [0,84 ; 0,97] | 0,077 [-0,149 ; 0,303] | -1,021 [-1,426 ; -0,616] | 1,175 [0,77 ; 1,58] | Yes |
| High CIBDAI | Expert 1 vs Expert 2 | 0,85 [0,7 ; 0,92] | -0,455 [-0,842 ; -0,067] | -2,595 [-3,282 ; -1,909] | 1,686 [1 ; 2,373] | No |
|  | Expert1 vs Non-expert 1 | 0,75 [0,57 ; 0,87] | 0,97 [0,559 ; 1,381] | -1,301 [-2,03 ; -0,573] | 3,241 [2,513 ; 3,969] | No |
|  | Expert 1 vs Non-expert 2 | 0,86 [0,73 ; 0,93] | -0,545 [-0,878 ; -0,213] | -2,385 [-2,974 ; -1,795] | 1,294 [0,704 ; 1,883] | No |
|  | Expert 2 vs Non-expert 1 | 0,80 [0,62 ; 0,9] | 0,515 [0,108 ; 0,923] | -1,737 [-2,459 ; -1,015] | 2,767 [2,045 ; 3,489] | No |
|  | Expert 2 vs Non-expert 2 | 0,89 [0,8 ; 0,95] | -0,091 [-0,403 ; 0,221] | -1,814 [-2,366 ; -1,262] | 1,632 [1,08 ; 2,184] | Yes |
|  | Non-expert 1 vs Non-expert 2 | 0,88 [0,76 ; 0,94] | 0,424 [0,143 ; 0,705] | -1,128 [-1,625 ; -0,63] | 1,976 [1,479 ; 2,474] | Yes |
| Low CCECAI | Expert 1 vs Expert 2 | 0,88 [0,76 ; 0,94] | -0,194 [-0,506 ; 0,117] | -1,998 [-2,548 ; -1,448] | 1,609 [1,059 ; 2,159] | Yes |
|  | Expert 1 vs Non-expert 1 | 0,78 [0,6 ; 0,88] | 0,278 [-0,107 ; 0,662] | -1,95 [-2,629 ; -1,271] | 2,506 [1,826 ; 3,185] | No |
|  | Expert 1 vs Non-expert 2 | 0,88 [0,77 ; 0,94] | -0,222 [-0,515 ; 0,071] | -1,919 [-2,436 ; -1,401] | 1,474 [0,957 ; 1,992] | Yes |
|  | Expert 2 vs Non-expert 1 | 0,84 [0,71 ; 0,92] | 0,083 [-0,254 ; 0,42] | -1,87 [-2,465 ; -1,274] | 2,036 [1,441 ; 2,632] | No |
|  | Expert 2 vs Non-expert 2 | 0,9 [0,8 ; 0,95] | -0,028 [-0,314 ; 0,258] | -1,683 [-2,188 ; -1,179] | 1,628 [1,123 ; 2,133] | Yes |
|  | Non-expert 1 vs Non-expert 2 | 0,92 [0,85 ; 0,96] | 0,056 [-0,172 ; 0,284] | -1,265 [-1,668 ; -0,862] | 1,376 [0,973 ; 1,779] | Yes |
| High CCECAI | Expert 1 vs Expert 2 | 0,86 [0,69 ; 0,94] | -0,435 [-0,92 ; 0,05] | -2,632 [-3,506 ; -1,758] | 1,763 [0,889 ; 2,636] | No |
|  | Expert1 vs Non-expert 1 | 0,81 [0,61 ; 0,92] | 1,087 [0,618 ; 1,555] | -1,037 [-1,881 ; -0,192] | 3,211 [2,366 ; 4,055] | No |
|  | Expert 1 vs Non-expert 2 | 0,89 [0,76 ; 0,95] | -0,609 [-0,971 ; -0,246] | -2,253 [-2,906 ; -1,599] | 1,035 [0,382 ; 1,689] | No |
|  | Expert 2 vs Non-expert 1 | 0,81 [0,58 ; 0,92] | 0,652 [0,09 ; 1,215] | -1,897 [-2,911 ; -0,883] | 3,201 [2,188 ; 4,215] | No |
|  | Expert 2 vs Non-expert 2 | 0,89 [0,76 ; 0,96] | -0,174 [-0,579 ; 0,231] | -2,01 [-2,74 ; -1,28] | 1,662 [0,932 ; 2,392] | No |
|  | Non-expert 1 vs Non-expert 2 | 0,91 [0,79 ; 0,96] | 0,478 [0,112 ; 0,844] | -1,18 [-1,839 ; -0,52] | 2,136 [1,477 ; 2,795] | No |
