# Supplementary material for: Towards a better understanding of clinical disease activity scores in dogs with chronic enteropathies
Source: Vet Q. 2025 Nov 3;45(1):2573447. doi: 10.1080/01652176.2025.2573447 (PMC12587788; doi:10.1080/01652176.2025.2573447)
Supplement: Supplementary file 1.docx [file TVEQ_A_2573447_SM4358.docx]

**Supplementary file 1.** Study of the intra-observer repeatability of CIBDAI, CCECAI and isolated variables for the pilot study. Data are provided with a 95% confidence interval. LOA: limit of agreement.

| **Score** | **Observer** | **Lin's concordance coefficient** | **Bias** | **Lower 95% LOA** | **Upper 95% LOA** | **Agreement** |
| --- | --- | --- | --- | --- | --- | --- |
| CIBDAI | Observer 1 | 0.98 [0.96; 0.99] | -0.17 [-0.36; 0.015] | -1.32 [-1.65; -1.0] | 0.98 [0.66; 1.31] | Yes |
|  | Observer 2 | 0.95 [0.91; 0.98] | 0.024 [-0.23; 0.27] | -1.52 [-1.96; -1.09] | 1.57 [1.13; 2.01] | Yes |
| CCECAI | Observer 1 | 0.96 [0.93; 0.98] | 0.049 [-0.215; 0.312] | -1.588 [-2.052; -1.125] | 1.686 [1.222; 2.149] | Yes |
|  | Observer 2 | 0.99 [0.97; 0.99] | -0.195 [-0.356; -0.034] | -1.196 [-1.48; -0.913] | 0.806 [0.523; 1.09] | Yes |
| Activity | Observer 1 | 1 | 0 | 0 | 0 | Yes |
|  | Observer 2 | 0.98 [0.96; 0.99] | -0.02 [-0.07; 0.02] | -0.33 [-0.42; -0.24] | 0.28 [0.2; 0.37] | Yes |
| Appetite | Observer 1 | 0.94 [0.89; 0.97] | -0.02 [-0.11; 0.06] | -0.56 [-0.71; -0.41] | 0.51 [0.36; 0.66] | Yes |
|  | Observer 2 | 0.90 [0.83; 0.95] | 0.02 [-0.09; 0.14] | -0.67 [-0.86; -0.47 | 0.72 [0.52; 0.91] | Yes |
| Vomiting | Observer 1 | 1 | 0 | 0 | 0 | Yes |
|  | Observer 2 | 0.91 [0.83; 0.95] | -0.07 [-0.18; 0.04] | -0.75 [-0.94; -0.56] | 0.6 [0.41; 0.8] | Yes |
| Fecal consistency | Observer 1 | 0.94 [0.89; 0.97] | 0 [-0.12; 0.12] | -0.76 [-0.97; -0.54] | 0.76 [0.54; 0.97] | Yes |
|  | Observer 2 | 0.87 [0.76; 0.93] | 0.05 [-0.12; 0.22] | -1.02 [-1.32; -0.72] | 1.12 [0.82; 1.42] | No |
| Frequency of defecation | Observer 1 | 0.94 [0.89; 0.97] | -0.15 [-0.26; -0.03] | -0.85 [-1.05; -0.65] | 0.56 [0.36; 0.75] | Yes |
|  | Observer 2 | 0.97 [0.94; 0.98] | 0 [-0.07; 0.07] | -0.44 [-0.56; -0.31] | 0.44 [0.31; 0.56] | Yes |
| Weight loss | Observer 1 | 1 | 0 | 0 | 0 | Yes |
|  | Observer 2 | 0.98 [0.97; 0.99] | 0.05 [-0.02; 0.12] | -0.38 [-0.5; -0.26] | 0.48 [0.36; 0.6] | Yes |
| Abdominal fluid and edema | Observer 1 | 1 | 0 | 0 | 0 | Yes |
|  | Observer 2 | 1 | 0 | 0 | 0 | Yes |
| Pruritus | Observer 1 | 1 | 0 | 0 | 0 | Yes |
|  | Observer 2 | 1 | 0 | 0 | 0 | Yes |
