# Supplementary material for: Towards a better understanding of clinical disease activity scores in dogs with chronic enteropathies
Source: Vet Q. 2025 Nov 3;45(1):2573447. doi: 10.1080/01652176.2025.2573447 (PMC12587788; doi:10.1080/01652176.2025.2573447)
Supplement: Supplementary file 4_AJ.docx [file TVEQ_A_2573447_SM4357.docx]

**Supplementary file 4.** Study of the inter-observer reproducibility of CIBDAI, CCECAI and isolated variables for the phase 2. Data are provided with a 95% confidence interval. LOA: limit of agreement.

| **Score** | **Observer** | **Lin's concordance coefficient** | **Bias** | **Lower 95% LOA** | **Upper 95% LOA** | **Agreement** |
| --- | --- | --- | --- | --- | --- | --- |
| CIBDAI | Expert 1 vs Expert 2 | 0.88 [0.81; 0.93] | 0.7119 [0.409 ; 1.014] | -1.563 [-2.09; -1.035] | 2.986 [2.459; 3.513] | No |
|  | Expert 1 vs Non-expert 1 | 0.9 [0.84; 0.94] | 0.3898 [0.103 ; 0.676] | -1.764 [-2.263; -1.265;] | 2.544 [2.044 : 3.044] | No |
|  | Expert 1 vs Non-expert 2 | 0.96 [0.93; 0.97] | 0.2712 [0.085; 0.458] | -1.13 [-1.455; -0.806] | 1.673 [1.348; 1.998] | Yes |
|  | Expert 2 vs Non-expert 1 | 0.93 [0.89; 0.96] | -0.322 [-0.585; -0.059] | -2.297 [-2.754; -1.839] | 1.653 [1.11; 1.95] | No |
|  | Expert 2 vs Non-expert 2 | 0.94 [0.89; 0.96] | -0.4407 [-0.679; -0.202] | -2.234 [-2.649; -1.818] | 1.352 [0.937; 1.768] | No |
|  | Non-expert 1 vs Non-expert 2 | 0.95 [0.91; 0.97] | -0.1186 [-0.346; 0.109] | -1.829 [-2.225; -1.433] | 1.592 [1.195; 1.988] | Yes |
| CCECAI | Expert 1 vs Expert 2 | 0.92 [0.86; 0.95] | 0.5932 [0.287; 0.9] | -1.712 [-2.246; -1.178] | 2.899 [2.364; 3.433] | No |
|  | Expert1 vs Non-expert 1 | 0.93 [0.88; 0.96] | 0.3051 [0.006; 0.604] | -1.946 [-2.468; -1.424] | 2.556 [2.034; 3.078] | No |
|  | Expert 1 vs Non-expert 2 | 0.97 [0.94; 0.98] | 0.2203 [0.02; 0.42] | -1.283 [-1.632; -0.935] | 1.724 [1.375; 2.072] | Yes |
|  | Expert 2 vs Non-expert 1 | 0.95 [0.92; 0.97] | -0.2881 [-0.549; -0.027] | -2.25 [-2.704; -1.795] | 1.674 [1.219; 2.128] | No |
|  | Expert 2 vs Non-expert 2 | 0.96 [0.93; 0.97] | -0.3729 [-0.599; -0.146] | -2.076 [-2.471; -1.681] | 1.33 [0.936; 1.725] | No |
|  | Non-expert 1 vs Non-expert 2 | 0.96 [0.94; 0.98] | -0.08475 [-0.313; 0.144] | -1.803 [-2.201; -1.405] | 1.634 [1.235; 2.032] | Yes |
| Activity | Expert 1 vs Expert 2 | 0.96 [0.93; 0.98] | 0.05085 [0.007; 0.109] | -0.3834 [-0.484; -0.283;] | 0.4851 [0.384; 0.586] | Yes |
|  | Expert1 vs Non-expert 1 | 0.96 [0.93; 0.97] | -0.01695 [-0.076 ; 0.042] | -0.4614 [-0.564; -0.358] | 0.4276 [0.325; 0.531] | Yes |
|  | Expert 1 vs Non-expert 2 | 0.99 [0.98; 0.99] | 0.01695 [-0.017; 0.051] | -0.2382 [-0.297; -0.179] | 0.2721 [0.213; 0.331] | Yes |
|  | Expert 2 vs Non-expert 1 | 0.92 [0.87; 0.95] | -0.0678 [-0.15 ; 0.014] | -0.6838 [-0.827; -0.541] | 0.5482 [0.405; 0.691] | Yes |
|  | Expert 2 vs Non-expert 2 | 0.97 [0.96; 0.98] | -0.0339 [-0.081; 0.014] | -0.3916 [-0.475; -0.309] | 0.3238 [0.241; 0.407] | Yes |
|  | Non-expert 1 vs Non-expert 2 | 0.97 [0.95; 0.98] | 0.0339 [-0.014; 0.081] | -0.3238 [-0.407; -0.241] | 0.3916 [0.309; 0.475] | Yes |
| Appetite | Expert 1 vs Expert 2 | 0.84 [0.75; 0.89] | 0.1017 [-0.003; 0.207] | -0.6869 [-0.87 ; -0.504] | 0.8903 [0.708; 1.073] | Yes |
|  | Expert1 vs Non-expert 1 | 0.85 [0.77; 0.91] | 0.0339 [-0.062 ; 0.13] | -0.6909 [-0.859; -0.523] | 0.7587 [0.591; 0.927] | Yes |
|  | Expert 1 vs Non-expert 2 | 0.94 [0.90; 0.96] | 0.01695 [-0.042; 0.076] | -0.4276 [-0.531; -0.325] | 0.4614 [0.358; 0.564] | Yes |
|  | Expert 2 vs Non-expert 1 | 0.86 [0.77; 0.91] | -0.0678 [-0.175; 0.039] | -0.8705 [-1.057; -0.684] | 0.7349 [0.549 ; 0.921] | Yes |
|  | Expert 2 vs Non-expert 2 | 0.89 [0.83; 0.93] | -0.08475 [-0.172; 0.003] | -0.7447 [-0.898; -0.592] | 0.5752 [0.422; 0.728] | Yes |
|  | Non-expert 1 vs Non-expert 2 | 0.91 [0.86; 0.95] | -0.01695 [-0.093; 0.059] | -0.5914 [-0.725; -0.458] | 0.5576 [0.424; 0.691] | Yes |
| Vomiting | Expert 1 vs Expert 2 | 0.75 [0.61; 0.84] | 0.1695 [0.04; 0.299] | -0.8039 [-1.029; -0.578] | 1.143 [0.917; 1.368] | No |
|  | Expert1 vs Non-expert 1 | 0.8 [0.69; 0.88] | 0.1695 [0.06; 0.279] | -0.6567 [-0.848; -0.465] | 0.9957 [0.804; 1.187] | No |
|  | Expert 1 vs Non-expert 2 | 0.95 [0.92; 0.97] | 0.05085 [-0.007; 0.109] | -0.3834 [-0.484; -0.283] | 0.4851 [0.384; 0.586] | Yes |
|  | Expert 2 vs Non-expert 1 | 0.88 [0.81; 0.93] | 0 [-0.097; 0.097] | -0.7279 [-0.897; -0.559] | 0.7279 [0.559; 0.897] | Yes |
|  | Expert 2 vs Non-expert 2 | 0.84 [0.74; 0.9] | -0.1186 [-0.228; -0.001] | -0.9394 [-1.13; -0.749] | 0.7021 [0.512; 0.892] | Yes |
|  | Non-expert 1 vs Non-expert 2 | 0.86 [0.77; 0.92] | -0.1186 [-0.216; -0.021] | -0.8542 [-1.025; -0.684] | 0.617 [0.446; 0.787] | Yes |
| Fecal consistency | Expert 1 vs Expert 2 | 0.87 [0.78; 0.92] | 0.2881 [0.169; 0.407] | -0.6072 [-0.815; -0.4] | 1.183 [0.976; 1.391] | No |
|  | Expert1 vs Non-expert 1 | 0.78 [0.64; 0.86] | 0.339 [0.174 ; 0.504] | -0.9012 [-1.189; -0.614] | 1.579 [1.292; 1.867] | No |
|  | Expert 1 vs Non-expert 2 | 0.93 [0.88; 0.96] | 0.1525 [0.058; 0.247] | -0.5582 [-0.723; -0.393] | 0.8633 [0.699; 1.028] | Yes |
|  | Expert 2 vs Non-expert 1 | 0.93 [0.88; 0.96] | 0.05085 [0.051; 0.153] | -0.7147 [-0.892; -0. 537] | 0.8164 [0.639; 0.994] | Yes |
|  | Expert 2 vs Non-expert 2 | 0.93 [0.89; 0.96] | -0.1356 [-0.226; -0.046] | -0.8124 [-0.969; -0.656] | 0.5412 [0.384; 0.698] | Yes |
|  | Non-expert 1 vs Non-expert 2 | 0.9 [0.83; 0.94] | -0.1864 [-0.3; -0.073] | -1.038 [-1.235; -0.841] | 0.6652 [0.468; 0.862] | No |
| Frequency of defecation | Expert 1 vs Expert 2 | 0.88 [0.81; 0.93] | -0.03448 [-0.164; 0.096] | -1.003 [-1.23; -0.777] | 0.9345 [0.708; 1.161] | No |
|  | Expert1 vs Non-expert 1 | 0.86 [0.78; 0.91] | -0.2241 [-0.364; -0.084] | -1.265 [-1.509; -1.022] | 0.8171 [0.574; 1.061] | No |
|  | Expert 1 vs Non-expert 2 | 0.95 [0.92; 0.97] | 0.01724 [-0.06; 0.095] | -0.5623 [-0.698; -0.427] | 0.5967 [0.461; 0.732] | Yes |
|  | Expert 2 vs Non-expert 1 | 0.92 [0.86; 0.95] | -0.1897 [-0.305; -0.075] | -1.047 [-1.248; -0.847] | 0.668 [0.467; 0.869] | No |
|  | Expert 2 vs Non-expert 2 | 0.93 [0.88; 0.96] | 0.05172 [-0.052; 0.155] | -0.7204 [-0.901; -0.54] | 0.8238 [0.643; 1.004] | Yes |
|  | Non-expert 1 vs Non-expert 2 | 0.88 [0.82; 0.93] | 0.2414 [0.118; 0.365] | -0.6809 [-0.897; -0.465] | 1.164 [0.948; 1.379] | No |
| Weight loss | Expert 1 vs Expert 2 | 0.83 [0.72; 0.89] | 0.1754 [0.001; 0.35] | -1.114 [-1.419; -0.81] | 1.465 [1.161; 1.77] | No |
|  | Expert1 vs Non-expert 1 | 0.89 [0.81; 0.93] | 0.1053 [-0.034; 0.244] | -0.92115 [-1.164; -0.679] | 1.132 [0.089; 1.374] | No |
|  | Expert 1 vs Non-expert 2 | 0.96 [0.93; 0.97] | 0.03509 [-0.051; 0.121] | -0.6027 [-0.753; -0.452] | 0.6729 [0.522; 0.823] | Yes |
|  | Expert 2 vs Non-expert 1 | 0.92 [0.86; 0.95] | -0.07018 [-0.192; 0.051] | -0.9668 [-1.179; -0.755] | 0.8265 [0.615; 1.038] | Yes |
|  | Expert 2 vs Non-expert 2 | 0.89 [0.81; 0.93] | -0.1404 [-0.286; 0.005] | -1.216 [-1.47; -0.962] | 0.9356 [0.682; 1.19] | No |
|  | Non-expert 1 vs Non-expert 2 | 0.93 [0.88; 0.96] | -0.07018 [-0.192; 0.051] | -0.9668 [-1.179; -0.755] | 0.8265 [0.615; 1.038] | Yes |
| Abdominal fluid and edema | Expert 1 vs Expert 2 | 0.98 [0.96; 0.99] | -0.01695 [-0.051; 0.017] | -0.2721[-0.331; -0.213] | 0.2382 [0.179; 0.297] | Yes |
|  | Expert1 vs Non-expert 1 | 0.91 [0.85; 0.95] | 0.0339 [-0.034; 0.102] | -0.4764 [-0.595; -0.358] | 0.5442 [0.426; 0.662] | Yes |
|  | Expert 1 vs Non-expert 2 | 0.98 [0.96; 0.99] | 0.01695 [-0.017; 0.051] | -0.2382 [-0.297; -0.179] | 0.2721 [0.213; 0.331] | Yes |
|  | Expert 2 vs Non-expert 1 | 0.93 [0.89; 0.96] | 0.05085 [0.007; 0.109] | -0.3834 [-0.484; -0.283] | 0.4851 [0.384; 0.586] | Yes |
|  | Expert 2 vs Non-expert 2 | 0.95 [0.92; 0.97] | 0.0339 [-0.014; 0.081] | -0.3238 [-0.407; -0.241] | 0.3916 [0.309; 0.475] | Yes |
|  | Non-expert 1 vs Non-expert 2 | 0.93 [0.89; 0.96] | -0.01695 [-0.076; 0.042] | -0.4614 [-0.564; -0.358] | 0.4276 [0.325; 0.531] | Yes |
| Pruritus | Expert 1 vs Expert 2 | 0.75 [0.61; 0.85] | -0.1017 [-0.207; 0.003] | -0.8903 [-1.073; -0.708] | 0.6869 [0.504; 0.87] | No |
|  | Expert1 vs Non-expert 1 | 0.76 [0.63; 0.85] | -0.1186 [-0.216; -0.021] | -0.8542 [-1.025; -0.684] | 0.617 [0.446; 0.787] | No |
|  | Expert 1 vs Non-expert 2 | 0.86 [0.77; 0.92] | -0.0678 [-0.15; 0.014] | -0.6838 [-0.827; -0.541] | 0.5482 [0.405; 0.691] | Yes |
|  | Expert 2 vs Non-expert 1 | 0.97 [0.95; 0.98] | -0.01695 [-0.051; 0.017] | -0.2721 [-0.331; -0.213] | 0.2382 [0.179; 0.297] | Yes |
|  | Expert 2 vs Non-expert 2 | 0.9 [0.84; 0.94] | 0.0339 [-0.034; 0.102] | -0.4764 [-0.595; -0.358] | 0.5442 [0.426; 0.662] | Yes |
|  | Non-expert 1 vs Non-expert 2 | 0.87 [0.79; 0.92] | 0.05085 [-0.024; 0.126] | -0.5158 [-0.647; -0.384] | 0.6175 [0.486; 0.749] | Yes |
