# Supplementary material for: Towards a better understanding of clinical disease activity scores in dogs with chronic enteropathies
Source: Vet Q. 2025 Nov 3;45(1):2573447. doi: 10.1080/01652176.2025.2573447 (PMC12587788; doi:10.1080/01652176.2025.2573447)
Supplement: Supplementary file 3_AJ.docx [file TVEQ_A_2573447_SM4356.docx]

**Supplementary file 3.** Study of the intra-observer repeatability of CIBDAI, CCECAI and isolated variables during phase 2. Data are provided with a 95% confidence interval. LOA: limit of agreement.

| **Score** | **Observer** | **Lin's concordance coefficient** | **Bias** | **Lower 95% LOA** | **Upper 95% LOA** |  | **Agreement** |
| --- | --- | --- | --- | --- | --- | --- | --- |
| CIBDAI | Expert 1 | 0.95 [0.91; 0.97] | 0.034 [-0.188; 0.255] | -1.633 [-2.019; -1.246] | 1.7 [1.314; 2.087] |  | Yes |
|  | Expert 2 | 0.96 [0.94; 0.98] | 0.017 [-0.185; 0.219] | -1.505 [-1.858; -1.152] | 1.539 [1.186; 1.892] |  | Yes |
|  | Non-expert 1 | 0.93 [0.89; 0.96] | -0.017 [-0.284; 0.25] | -2.027 [-2.492; -1.561] | 1.993 [1.527; 2.459] |  | No |
|  | Non-expert 2 | 0.99 [0.98; 0.99] | -0.034 [-0.152; 0.084] | -0.923 [-1.129; -0.717] | 0.855 [0.649; 1.061] |  | Yes |
| CCECAI | Expert 1 | 0.96 [0.93; 0.97] | 0.051 [-0.188; 0.29] | -1.748 [-2.165; -1.331] | 1.85 [1.433; 2.266] |  | Yes |
|  | Expert 2 | 0.97 [0.95; 0.98] | 0 [-0.205; 0.205] | -1.544 [-1.902; -1.186] | 1.544 [1.186; 1.902] |  | Yes |
|  | Non-expert 1 | 0.95 [0.91; 0.97] | -0.034 [-0.308; 0.24] | -2.092 [-2.569; -1.615] | 2.024 [1.547; 2.501] |  | No |
|  | Non-expert 2 | 0.99 [0.98; 0.99] | -0.051 [-0.173; 0.072] | -0.973 [-1.187; -0.76] | 0.872 [0.658; 1.085] |  | Yes |
| Activity | Expert 1 | 0.97 [0.95; 0.98] | 0 [-0.048; 0.048] | -0.364 [-0.448; -0.28] | 0.364 [0.28; 0.448] |  | Yes |
|  | Expert 2 | 0.97 [0.96; 0.98] | -0.034 [0.081; 0.014] | -0.392 [-0.475; -0.309] | 0.324 [0.241; 0.407] |  | Yes |
|  | Non-expert 1 | 0.97 [0.95; 0.98] | 0.034 [-0.014; 0.081] | -0.324 [-0.407; -0.241] | 0.392 [0.309; 0.475] |  | Yes |
|  | Non-expert 2 | 1 | 0 [0; 0] | 0 [0; 0] | 0 [0; 0] |  | Yes |
| Appetite | Expert 1 | 0.87 [0.79; 0.92] | 0 [-0.084; 0.084] | -0.63 [-0.776; -0.484] | 0.63 [0.484; 0.776] |  | Yes |
|  | Expert 2 | 0.96 [0.94; 0.98] | 0.051 [-0.007; 0.109] | -0.383 [-0.484; -0.283] | 0.485 [0.384; 0.586] |  | Yes |
|  | Non-expert 1 | 0.88 [0.8; 0.93] | 0.034 [-0.062; 0.13] | -0.691 [-0.859; -0.523] | 0.759 [0.591; 0.927] |  | Yes |
|  | Non-expert 2 | 0.96 [0.94; 0.98] | 0.034 [-0.014; 0.081] | -0.324 [-0.407; -0.241] | 0.392 [0.309; 0.475] |  | Yes |
| Vomiting | Expert 1 | 0.9 [0.84; 0.93] | 0.085 [-0.003; 0.172] | -0.575 [-0.728; -0.422] | 0.745 [0.592; 0.898] |  | Yes |
|  | Expert 2 | 0.85 [0.76; 0.91] | 0.102 [-0.014; 0.217] | -0.767 [-0.968; -0.566] | 0.97 [0.769; 1.172] |  | Yes |
|  | Non-expert 1 | 0.75 [0.61; 0.84] | -0.119 [-0.265; 0.027] | -1.216 [-1.47; -0.961] | 0.978 [0.724; 1.233] |  | No |
|  | Non-expert 2 | 1 | 0 [0; 0] | 0 [0; 0] | 0 [0; 0] |  | Yes |
| Fecal consistency | Expert 1 | 0.92 [0.87; 0.95] | 0 [-0.108;0.108] | -0.814 [-1.002; -0.625] | 0.814 [0.625; 1.022] |  | Yes |
|  | Expert 2 | 0.94 [0.9; 0.97] | -0.119 [-0.204; -0.034] | -0.758 [-0.906; -0.61] | 0.521 [0.372; 0.669] |  | Yes |
|  | Non-expert 1 | 0.94 [0.9; 0.96] | -0.068 [-0.163; 0.027] | -0.783 [-0.949; -0.617] | 0.648 [0.482; 0.813] |  | Yes |
|  | Non-expert 2 | 0.97 [0.96; 0.98] | -0.017 [-0.076; 0.042] | -0.461 [-0.564; -0.358] | 0.428 [0.325; 0.531] |  | Yes |
| Frequency of defecation | Expert 1 | 0.96 [0.93; 0.98] | -0.034 [-0.104; 0.035] | -0.549 [-0.67; -0.429] | 0.48 [0.36; 0.601] |  | Yes |
|  | Expert 2 | 0.92 [0.87; 0.95] | 0.034 [-0.075; 0.144] | -0.784 [-0.975; -0.592] | 0.853 [0.661; 1.044] |  | Yes |
|  | Non-expert 1 | 0.85 [0.76; 0.91] | 0.224 [0.076; 0.372] | -0.88 [-1.138; -0.622] | 1.328 [1.07; 1.586] |  | No |
|  | Non-expert 2 | 0.97 [0.95; 0.97] | -0.017 [-0.077; 0.043] | -0.466 [-0.57; -0.361] | 0.431 [0.326; 0.536] |  | Yes |
| Weight loss | Expert 1 | 0.93 [0.89; 0.96] | -0.017 [-0.076; 0.042] | -0.461 [-0.564; -0.358] | 0.428 [0.325; 0.531] |  | Yes |
|  | Expert 2 | 0.95 [0.91; 0.97] | -0.053 [-0.158; 0.053] | -0.831 [-1.015; -0.648] | 0.726 [0.542; 0.91] |  | Yes |
|  | Non-expert 1 | 0.85 [0.76; 0.91] | 0.224 [0.076; 0.372] | -0.88 [-1.138; -0.622] | 1.328 [1.07; 1.586] |  | No |
|  | Non-expert 2 | 0.99 [0.98; 0.99] | -0.035 [-0.084; 0.014] | -0.399 [-0.485; -0.313] | 0.329 [0.243; 0.415] |  | Yes |
| Abdominal fluid and edema | Expert 1 | 0.98 [0.96; 0.99] | 0.017 [-0.017; 0.051] | -0.238 [-0.297; -0.579] | 0.272 [0.213; 0.331] |  | Yes |
|  | Expert 2 | 1 | 0 [0; 0] | 0 [0; 0] | 0 [0; 0] |  | Yes |
|  | Non-expert 1 | 0.93 [0.89; 0.96] | -0.017 [-0.076; 0.042] | -0.461 [-0.564; -0.358] | 0.428 [0.325; 0.531] |  | Yes |
|  | Non-expert 2 | 1 | 0 [0; 0] | 0 [0; 0] | 0 [0; 0] |  | Yes |
| Pruritus | Expert 1 | 0.8 [0.69; 0.88] | 0 [-0.097; 0.097] | -0.728 [-0.897; -0.559] | 0.728 [0.559; 0.897] |  | No |
|  | Expert 2 | 0.97 [0.95; 0.98] | -0.017 [-0.051; 0.017] | -0.272 [-0.331; -0.213] | 0.238 [0.179; 0.297] |  | Yes |
|  | Non-expert 1 | 1 | 0 [0; 0] | 0 [0; 0] | 0 [0; 0] |  | Yes |
|  | Non-expert 2 | 0.98 [0.96; 0.99] | -0.017 [-0.051; 0.017] | -0.272 [-0.331; -0.213] | 0.238 [0.179; 0.297] |  | Yes |
