# Supplementary material for: Towards a better understanding of clinical disease activity scores in dogs with chronic enteropathies
Source: Vet Q. 2025 Nov 3;45(1):2573447. doi: 10.1080/01652176.2025.2573447 (PMC12587788; doi:10.1080/01652176.2025.2573447)
Supplement: Supplementary file 2.docx [file TVEQ_A_2573447_SM4355.docx]

**Supplementary file 2.** Study of the inter-observer reproducibility of CIBDAI, CCECAI and isolated variables for the pilot study. Data are provided with a 95% confidence interval. LOA: limit of agreement.

| **Score** | **Lin's concordance coefficient** | **Bias** | **Lower 95% LOA** | **Upper 95% LOA** | **Agreement** |
| --- | --- | --- | --- | --- | --- |
| CIBDAI | 0.88 [0.78; 0.93] | 0.17 [-0.26; 0.60] | -2.49 [-3.25; -1.74] | 2.83 [2.08; 3.59] | No |
| CCECAI | 0.91 [0.83; 0.95] | 0.049 [-0.386; 0.484] | -2.651 [-3.416; -1.886] | 2.749 [1.984; 3.513] | No |
| Activity | 0.98 [0.96; 0.99] | 0.02 [-0.02; 0.07] | -0.28 [-0.37; -0.2] | 0.33 [0.24; 0.42] | Yes |
| Appetite | 0.83 [0.7; 0.91] | 0.02 [-0.13; 0.17] | -0.90 [-1.17; -0.64] | 0.95 [0.69; 1.22] | Yes |
| Vomiting | 0.87 [0.77; 0.93] | 0.15 [0.01; 0.28] | -0.68 [-0.91; -0.45 | 0.97 [0.74; 1.21] | Yes |
| Fecal consistency | 0.85 [0.73; 0.92] | 0.15 [-0.03; 0.33] | -0.98 [-1.29; -0.66] | 1.27 [0.95; 1.59] | No |
| Frequency of defecation | 0.86 [0.75; 0.92] | -0.20 [-0.36; -0.03] | -1.20 [-1.48; -0.91] | 0.81 [0.52; 1.09] | No |
| Weight loss | 0.80 [0.66; 0.89] | 0.07 [-0.15; 0.3] | -1.34 [-1.74; -0.94] | 1.49 [1.09; 1.89] | No |
| Abdominal fluid and edema | 0.97 [0.94; 0.98] | -0.02 [-0.07; 0.02] | -0.33 [-0.42; -0.24] | 0.28 [0.2; 0.37] | Yes |
| Pruritus | 1 | 0 | 0 | 0 | Yes |
